# Measurement of Radial Elasticity and Original Height of DNA Duplex Using Tapping-Mode Atomic Force Microscopy

**DOI:** 10.3390/nano9040561

**Published:** 2019-04-06

**Authors:** Longhai Li, Xu Zhang, Hongfei Wang, Qian Lang, Haitao Chen, Lian Qing Liu

**Affiliations:** 1 College of Engineering, Northeast Agricultural University, Harbin 150030, China; lilonghai@neau.edu.cn (L.L.); zx957xz@163.com (X.Z.); CH_WHF@163.com (H.W.); langqian4521@126.com (Q.L.); 2 State Key Laboratory of Robotics, Shenyang Institute of Automation, Chinese Academy of Sciences, Shenyang 110016, China

**Keywords:** DNA duplex, Young’s modulus, AFM, tapping mode

## Abstract

Atomic force microscopy (AFM) can characterize nanomaterial elasticity. However, some one-dimensional nanomaterials, such as DNA, are too small to locate with an AFM tip because of thermal drift and the nonlinearity of piezoelectric actuators. In this study, we propose a novel approach to address the shortcomings of AFM and obtain the radial Young’s modulus of a DNA duplex. The elastic properties are evaluated by combining physical calculations and measured experimental results. The initial elasticity of the DNA is first assumed; based on tapping-mode scanning images and tip–sample interaction force simulations, the calculated elastic modulus is extracted. By minimizing the error between the assumed and experimental values, the extracted elasticity is assigned as the actual modulus for the material. Furthermore, tapping-mode image scanning avoids the necessity of locating the probe exactly on the target sample. In addition to elasticity measurements, the deformation caused by the tapping force from the AFM tip is compensated and the original height of the DNA is calculated. The results show that the radial compressive Young’s modulus of DNA is 125–150 MPa under a tapping force of 0.5–1.3 nN; its original height is 1.9 nm. This approach can be applied to the measurement of other nanomaterials.

## 1. Introduction

After the DNA origami technique was developed [1], DNA and assembled structures [2,3,4,5] have been suggested as connections for other nanostructures to construct high-order devices and systems [6,7] because they offer high yield and strong attachment to metals [8], semiconductors [9], and biomaterials [10,11]. To stabilize such systems and devices, understanding the elastic properties of DNA duplexes is necessary. Atomic force microscopy (AFM) [12], used to characterize the geometric [13] and mechanical properties of nanomaterials [14,15,16,17,18,19], was the primary tool used to measure the axial Young’s modulus of DNA via the force–distance curve method [20,21,22,23]. However, obtaining the radial Young’s modulus of such a one-dimensional (1D) nanomaterial was impeded by the difficulty of placing the probe exactly on such a small specimen, particularly considering thermal drift and the nonlinearity of the piezoelectric actuators used in AFM probe tips. Vibrating scanning polarization force microscopy (VSPFM) [24], invented by Hu et al., measures charged tip-induced dielectric polarization forces on sample surfaces; it has been used to measure the radial elasticity of λ-DNA. The compression elasticity of a single-strand DNA (ssDNA) chain varies with the tip–sample interaction force, which is ≈20–70 MPa under a tapping force of 0.4 nN. With increasing tapping force, the elastic modulus increases to more than 100 MPa. The VSPFM method is effective in air, but not in a liquid environment. Pang et al. [25] calculated the compressive Young’s modulus of ssDNA in the radial direction with the Hertz model by using the theoretical height of DNA (2 nm) and assuming that all cantilever bending energy was transferred to DNA deformation. However, some physical phenomena, such as thermal distortion and plastic deformation, consume some energy, inducing errors. Furthermore, this method is not applicable for nanowires of unknown original heights. 

In this study, by simulating tip–sample interactions and measuring the experimental height of a DNA duplex, a new assumption–calculation method (ACM), which minimizes the error between the assumed and calculated Young’s moduli of DNA, is developed to characterize the radial Young’s modulus of DNA. Moreover, the original height of a DNA duplex was calculated by compensating for the indentation caused by the tapping force from the AFM probe tip. 

## 2. Materials and Methods

### 2.1. Assumption–Calculation Method (ACM)

In tapping-mode AFM, the dynamics of the AFM tip can be described using the point-mass model [26], as shown in Equation (1):

(1) $$\frac{k}{\omega_{o}^{2}}\ddot{x}+\frac{k}{Q\omega_{o}}\dot{x}+kx=\frac{kA_{o}}{Q}cos(\omega_{o}t)+F_{ts}$$

where *x* is the AFM tip position relative to substrate surface, *k* is the spring constant of the AFM cantilever, $\omega_{o}$ is the resonant frequency of the cantilever, $kA_{o}/Q$ is the external force acting on the cantilever from the crystal oscillator that drives the tip vibration with the free amplitude *A_o_*, and Q  is the quality factor of the cantilever. *F_ts_* is the tip–sample interaction force, which is described by the Derjaguin–Müller–Toporov (DMT) model in Equation (2):

(2) $$F_{ts}=\{\begin{array}{c} -\frac{HR}{6a_{o}^{2}}<d\\ -\frac{HR}{6a_{o}^{2}}+\frac{4}{3}E^{*}\sqrt{R}{\left(a_{o}-d\right)}^{\frac{3}{2}}a_{o}\geq d \end{array}$$

where *H*, *R*, *a_o_*, and *d* are the Hamaker constant, tip radius, intermolecular distance, and indentation of the sample surface caused by the AFM tip, respectively. E* is the effective elastic modulus of the tip and sample, which is expressed using Equation (3):

(3) $$E^{*}={\left(\frac{v_{1}}{E_{1}}+\frac{v_{2}}{E_{2}}\right)}^{-1}$$

where *v*_1_ and *v*_2_ are the Poisson ratios of the sample and AFM tip, respectively; $E_{1}$ and $E_{2}$ are the Young’s moduli of the sample and AFM tip, respectively. 

**Figure 1:**
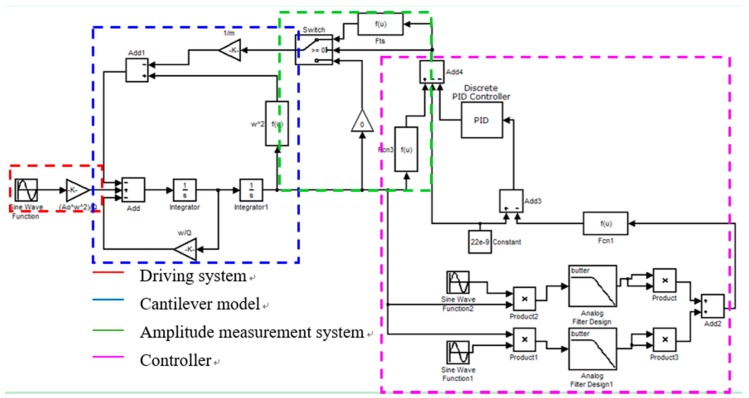
Key components of numerical simulations, including the driving system, cantilever model, amplitude measurement, and feedback loop of the PID controller.

According to the dynamic Equation (1), the scanning process can be simulated with MATLAB software after providing the parameters mentioned above [27]. As shown in Figure 1, the numerical simulation schematic includes the cantilever drive, a cantilever model containing the tip–sample force model and the amplitude measurement model, and a feedback loop with a proportional-integral-derivative (PID) controller. From the simulation, we can obtain information on tip vibration, tip–sample interaction, and sample indentation. The time-varying tapping process image indicates that the maximum indentation *d* of the sample occurs at the peak tapping force in one cycle when the tip approaches the bottom.

**Figure 2:**
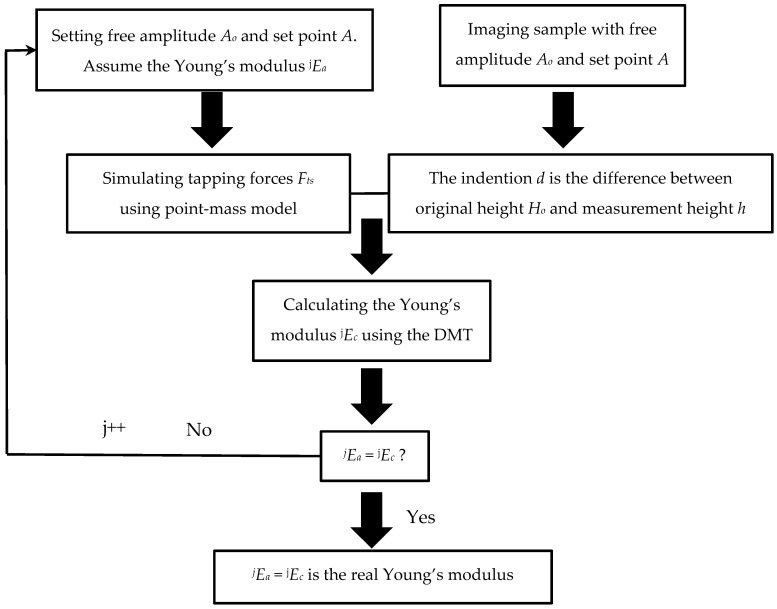
Flowchart of the assumption–calculation method. *j* represents the assumed Young’s modulus used in simulation.

To obtain the physical properties of unfamiliar materials, we can assume the Young’s modulus as *E_a_* and simulate the peak tapping force *F_max_* at different free amplitudes *A_o_* and the set point amplitude *A*. Simultaneously, scanning experiments of the sample can be implemented with AFM under the same parameters as those used in the simulation, including the scanning parameters and the tip and sample properties. After scanning, the measured height *h* of the sample is obtained for different free amplitudes and set point amplitudes corresponding with the simulation. However, the measured height *h* is not the real sample height because the indentation *d* is induced under tip contact with the sample at the tapping force. Therefore, the original height *H_o_* is the sum of the indentation *d* and the measurement height *h*, i.e., *H_o_* = *d* + *h*. Using the peak tapping force *F_max_*, original height *H_o_*, and other parameters in the DMT model, we can obtain the calculated Young’s modulus *E_c_*. In comparing *E_c_* with *E_a_*, if the assumed Young’s modulus *E_a_* is the real elastic modulus of the sample, it should be equal to the calculated Young’s modulus *E_c_*. If *E_a_* is not equal to *E_c_*, we assume another Young’s modulus and repeat the same steps mentioned above until reaching *E_c_* = *E_a_*, which is the real Young’s modulus of the sample. The specific steps of the ACM are shown in Figure 2.

### 2.2. AFM Experiments

#### 2.2.1. Sample Preparation

ChR plasmid DNA (878 ng μL^−1^), purchased from Takara Company (Dalian, China), was diluted to 0.3 μmol L^−1^ with a buffer solution comprising 20 mM Tris-HCl at pH 7.6, 1 mM ethylenediaminetetraacetic acid (EDTA), and 10–20 mM MgCl_2_ (TE/Mg^2+^, Takara Company). A droplet of 3 μL DNA solution was dropped on freshly cleaved mica and held for 5 min. The mica was then dried in compressed air for 3 min before 15 μL TE/Mg^2+^ buffer solution was dropped on the dried sample. The sample was then placed on the AFM stage for imaging.

#### 2.2.2. AFM Images

**Figure 3:**
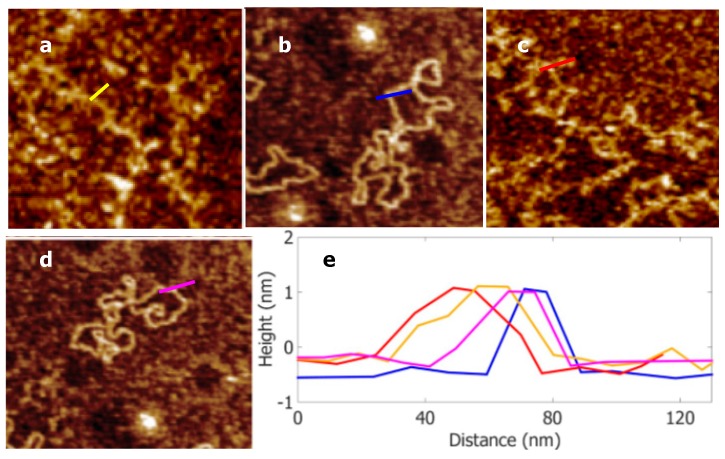
AFM images of DNA duplexes on mica surface. (**a**) Free amplitude was 38 nm, set point was 21 nm, and measured height was 1.1 nm. (**b**) Free amplitude was 16 nm, set point was 14 nm, and measured height was 1.5 nm. (**c**) Free amplitude was 30 nm, set point was 21 nm, and measured height was 1.3 nm. (**d**) Free amplitude was 22 nm, set point was 17 nm, and measured height was 1.4 nm. (**e**) Section curves taken along the lines in (**a**–**d**), as indicated by color.

The images of samples in a liquid environment were obtained using a Multimode AFM (Bruker, Santa Barbara, CA, USA) with an E scanner head in tapping mode. A silicon nitride probe (DNP-10, Bruker) was selected for scanning; it had quality factor *Q* of 20, resonant frequency *f_o_* of 8.5 kHz, tip radius *R* of 3 nm, Young’s modulus *E_tip_* of 310 GPa, and Poisson ratio *v* of 0.3. The spring constant *k* was measured at 0.12 N/m with the thermal tune method [28] in NanoScope software (V1.5, Bruker). The intermolecular distance *a_o_* was estimated to be 2 × 10^−10^ m [29], and the Hamaker constant *H* was calculated to be 2 × 10^−20^ J [29,30] with $H\approx \left(\sqrt{H_{tt}}-\sqrt{H_{ll}}\right)\times \left(\sqrt{H_{DD}}-\sqrt{H_{ll}}\right)$, where $H_{tt}$, $H_{ll}\;$, and $H_{DD}\;$ are the Hamaker constants of the tip, liquid, and DNA, respectively. Here, we replaced the *H* of DNA with that of proteins. The matrix **A^f^_s_** provides the parameters for tip amplitude used in the experiments, where the superscript *f* depicts the free amplitude of the cantilever and the subscript *s* indicates the set point amplitude used in scanning (nm). After scanning, the measurement height **H^f^_s_** under **A^f^_s_** was calculated from each scanning picture by averaging the values *^i^H^f^_s_* of five DNA duplexes, which were used to calculate the Young’s modulus of DNA; the superscript *i* depicts the number of DNA duplexes (1–5). During scanning, 512 × 512 sample points were used to obtain high-resolution AFM images; Figure 3 shows examples of AFM images and height measurements. The matrix **H^f^_s_** provides the measured heights of the DNA duplexes.

$$A_{s}^{f}=\left[\begin{array}{ccccc} A_{25}^{38} & A_{23}^{38} & A_{21}^{38} & A_{19}^{38} & A_{17}^{38}\\ A_{21}^{30} & A_{19}^{30} & A_{17}^{30} & A_{15}^{30} & A_{13}^{30}\\ A_{17}^{22} & A_{15}^{22} & A_{13}^{22} & A_{11}^{22} & A_{9}^{22}\\ A_{14}^{16} & A_{12}^{16} & A_{10}^{16} & A_{8}^{16} & A_{6}^{16} \end{array}\right]$$

$$H_{s}^{f}=\left[\begin{array}{ccccc} H_{25}^{38} & H_{23}^{38} & H_{21}^{38} & H_{19}^{38} & H_{17}^{38}\\ H_{21}^{30} & H_{19}^{30} & H_{17}^{30} & H_{15}^{30} & H_{13}^{30}\\ H_{17}^{22} & H_{15}^{22} & H_{13}^{22} & H_{11}^{22} & H_{9}^{22}\\ H_{14}^{16} & H_{12}^{16} & H_{10}^{16} & H_{8}^{16} & H_{6}^{16} \end{array}\right]=\left[\begin{array}{ccccc} 1.3 & 1.2 & 1.1 & 1.1 & 1.1\\ 1.3 & 1.3 & 1.2 & 1.1 & 1.2\\ 1.4 & 1.3 & 1.3 & 1.2 & 1.3\\ 1.5 & 1.4 & 1.3 & 1.2 & 1.3 \end{array}\right]$$

## 3. Results and Discussion

**Figure 4:**
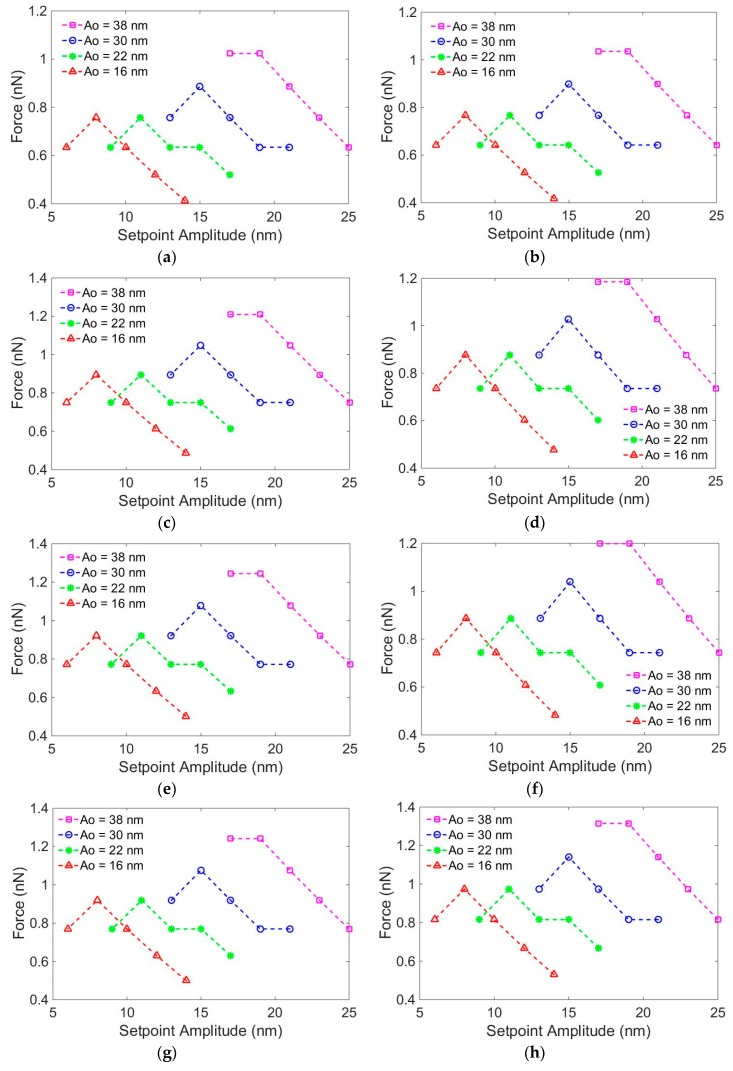

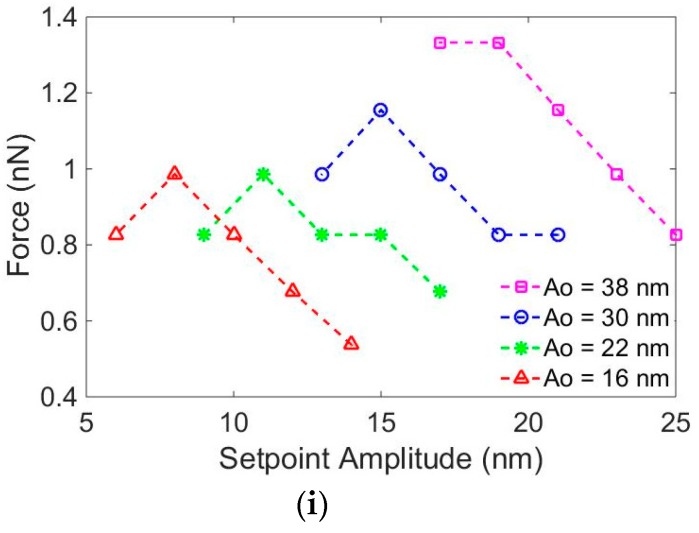
Simulating the maximum tip–sample interaction forces with different assumed Young’s moduli and other parameters set equal to those used in the experiments: *E_a_* = (**a**) 50 MPa, (**b**) 75 MPa, (**c**) 100 MPa, (**d**) 125 MPa, (**e**) 150 MPa, (**f**) 175 MPa, (**g**) 200 MPa, (**h**) 225 MPa, and (**i**) 250 MPa.

With the measurement height of DNA duplexes $H_{s}^{f}$, the maximal sample indentation $d_{s}^{f}$ from the tip–sample interaction under the tip contact with the sample during scanning can be calculated using $d_{s}^{f}=H_{o}-H_{s}^{f}$. Using the scanning amplitudes $A_{s}^{f}$ and the mechanical properties of the DNP AFM tip mentioned above, the tip–sample interaction force Fjsf could be simulated using the point-mass model (Equation (1)) with the assumed Young’s modulus *E_a_* of 50–250 MPa at 25-MPa increments. The maximal tapping force Fjsf at the free amplitude and set-point amplitude of $A_{s}^{f}\;$ are shown in Figure 4, where the superscript *j* is the assumed Young’s modulus used in the simulation.

By using the simulated tapping force Fjsf and the indentation of sample $d_{s}^{f}=H_{o}-H_{s}^{f}$ in the DMT model (Equation (2)), we obtain Equation (4):

(4) Fjsf=−HR6a2+43(v1Ejc+v2E2)−1R12(ao−(Hoj−Hsf))32

where Ejc represents the calculated Young’s modulus corresponding to the assumed Young’s modulus Eja. $H_{o}^{j}$ is the calculated original height. From Equation (4), for each matrix **^j^F_s_^f^**, we obtained the calculated Young’s modulus matrix Ejc and the original height matrix $H_{o}^{j}$.

Ecj=[E2538jE2338jE2138jE1938jE1738jE2130jE1930jE1730jE1530jE1330jE1722jE1522jE1322jE1122jE922jE1416jE1216jE1016jE816jE616j] Hoj=[Hoj2538Hoj2338Hoj2138Hoj1938Hoj1738Hoj2130Hoj1930Hoj1730Hoj1530Hoj1330Hoj1722Hoj1530Hoj1330Hoj1130Hoj930Hoj1416Hoj1216Hoj1016Hoj816Hoj616]

For each assumed Young’s modulus Eja, we obtained the calculated Young’s modulus and its standard deviation by averaging the calculated Young’s modulus matrix Ejc**.** The same information for original height was obtained via the same method. Thus, the calculated Young’s modulus matrix $E_{c}$ and the original height matrix $H_{o}$ were obtained.

Ec=[E50cE75cE100cE125cE150cE175cE200cE225cE250c]

$$H_{o}=\left[\begin{array}{ccccccccc} H_{o}^{50} & H_{o}^{75} & H_{o}^{100} & H_{o}^{125} & H_{o}^{150} & H_{o}^{175} & H_{o}^{200} & H_{o}^{225} & H_{o}^{250} \end{array}\right]$$

**Figure 5:**
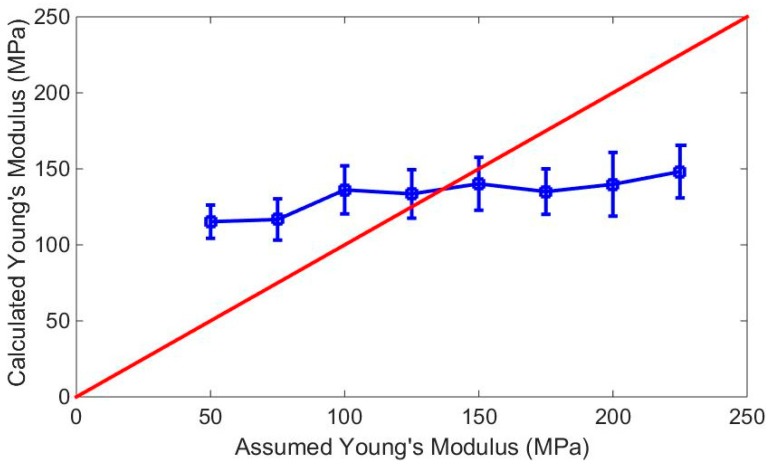
Elasticity of DNA. Red line: *x* = *y*. Blue line: *E_c_*.

The tapping forces Fsfj used in Equation (4) were simulated according to the assumed Young’s moduli Eja, and the measurement heights $H_{s}^{f}\;$ were obtained experimentally under parameters equal to those in the simulation, except for the Young’s modulus. If the simulated tapping force obtained with the assumed Young’s modulus was not equal to the experimental tapping force caused by the real Young’s modulus, the calculated Young’s modulus Ejc solved using Equation (4) with the simulated tapping force and measurement height was not equal to the assumed Young’s modulus. Therefore, neither the assumed nor calculated Young’s modulus were the real elasticity of the sample. Otherwise, if the assumed value was equal to the calculated one, both of them were the real Young’s modulus of sample. We plot the calculated Young’s moduli points in Figure 5. The horizontal and vertical axes represent the assumed and calculated Young’s modulus values, respectively. The points on the red line indicate *x* = *y* and the points on the blue curve are the calculated Young’s moduli values from our experiments. According to our method, the points at the intersection of the red line *x* = *y* and the blue curve are meaningful for us because they denote Eja=Ejc , which indicates a matching of the assumed and calculated Young’s moduli. From Figure 5, the red line intersects with the E125a and E150a  columns. This indicates that the radial Young’s modulus of the DNA duplex was between 125 MPa and 150 MPa, and that the tapping force value was between 0.5 nN and 1.3 nN. The original height of the DNA duplex was approximately 1.9 nm, which is smaller than the theoretical value; this may be attributed to the electrostatic force between the DNA and the mica surface in liquid.

The stiffness of the DNA duplex obtained with our method was higher than that measured by other researchers [31], which is about 5–30 MPa in the force range of ≈60–160 pN. This discrepancy is likely from two primary reasons. One is that other studies used weaker acting forces than that in our experiments. Furthermore, the change in the mechanical properties of DNA with different loading forces has been demonstrated. Another reason is the stronger substrate effect experienced by the samples in our experiments using the mica surface, as opposed to those experienced with DNA origami surfaces in Reference [31]. 

Obviously, by measuring the Young’s modulus of the DNA duplex with our approach, both the mechanical property and original height could be obtained. However, the values under the pico-newton scale of tapping forces would be difficult to measure because small tapping forces cannot obtain quality scanning AFM images in the tapping mode. However, multi-harmonic atomic force microscopy is a good choice for obtaining the mechanical properties at the pico-newton scale [32,33,34]. Furthermore, our approach is more suitable for measuring the mechanical properties of soft nanomaterials because the measured height values of hard materials differ slightly under similar tapping forces.

## 4. Conclusions

In this study, an AFM tapping-mode scanning-based method was proposed to measure the radial elasticity and original height of DNA duplexes through a combination of theoretical calculation and experimental measurements. These are suitable for materials that cannot be characterized by the force curve method. With the proposed method, the Young’s modulus was obtained not by performing a shot-plot curve exactly over the sample surface, but through the scanning of several images with assorted free and set-point amplitudes. The critical prerequisite for the ability of accurately positioning the tip is thus avoided. The indentation of the sample caused by the tapping force exerted by the AFM probe tip can be compensated for, thus allowing for calculation of the original height of DNA. The experimental results show that the Young’s modulus of DNA was 125–150 MPa under a tapping force of 0.5–1.3 nN, while the original height was 1.9 nm. These results agree with other DNA elasticity measurements obtained using other methods. The obtained Young’s modulus and original height of the DNA duplexes demonstrate the effectiveness and validity of the proposed method. The presented method is simpler and applicable to other nanomaterials.
